# Exploring the Plasma Fatty Acid Signature of Primary Aldosteronism: Comparison with Essential Hypertension and Longitudinal Therapy Effects

**DOI:** 10.3390/diagnostics15192465

**Published:** 2025-09-26

**Authors:** Gabriele Mango, Annalisa Castagna, Patrizia Pattini, Sergio De Marchi, Carlotta Spillere, Khulah Sadia, Francesca Begali, Sara Moruzzi, Nicola Martinelli, Luigi Marzano, Simonetta Friso, Francesca Pizzolo

**Affiliations:** 1 Department of Medicine, University of Verona, 37134 Verona, Italy; gabriele.mango@univr.it (G.M.); patrizia.pattini@univr.it (P.P.); carlotta.spillere@studenti.univr.it (C.S.); khulah.sadia@univr.it (K.S.); francesca.begali_01@studenti.univr.it (F.B.); nicola.martinelli@univr.it (N.M.); simonetta.friso@univr.it (S.F.); francesca.pizzolo@univr.it (F.P.); 2 Department of Angiology, Integrated University Hospital of Verona, 37134 Verona, Italy; sergio.demarchi@univr.it; 3 Unit of Internal Medicine B, Department of Medicine, University of Verona, Integrated University Hospital of Verona, 37134 Verona, Italy; sara.moruzzi@aovr.veneto.it (S.M.); luigi.marzano@aovr.veneto.it (L.M.)

**Keywords:** fatty acids, primary aldosteronism, essential hypertension, mineralocorticoid receptor antagonists, adrenalectomy

## Abstract

**Background/Objectives**: Fatty acids (FAs) play crucial roles in human physiology, and their levels have been associated with hypertension, although with inconsistent findings. Primary Aldosteronism (PA), a common, often underdiagnosed form of secondary hypertension, carries a higher risk of organ damage compared to essential hypertension (EH). This study aimed to compare plasma FA profiles of patients with unilateral PA and EH and explore the impact of therapies. **Methods**: Participants were recruited at the Hypertension Unit of Verona University Hospital. PA diagnosis/subtype was confirmed according to guidelines. Blood samples were collected at enrollment and at follow-up (after treatment with a mineralocorticoid receptor antagonist (MRA) and adrenalectomy). Plasma long- and very-long-chain FAs were extracted and analyzed using gas chromatography. **Results**: Each sample was assessed for a panel of 19 selected FA species. Compared to EH (*n* = 60), PA patients (*n* = 22) exhibited lower plasma levels of behenic acid (*p* = 0.03), total monounsaturated fatty acids (*p* = 0.02), specifically palmitoleic (*p* = 0.005) and erucic acids (*p* = 0.02), and higher levels of ω6 polyunsaturated fatty acids (PUFAs, *p* = 0.02). Longitudinal analysis in PA patients showed that MRAs decreased total saturated FAs (*p*_ADJ_ = 0.01) and increased total PUFAs (*p*_ADJ_ = 0.006), and these changes were largely maintained even after adrenalectomy. **Conclusions**: This pilot study reveals significant alterations in the plasma FA profiles of PA patients compared to EH, suggesting a more prominent inflammatory state in PA. Both pharmacological and surgical interventions induced a positive shift in the FA profile of PA patients. These findings highlight the potential of FAs as biomarkers for PA risk stratification and may offer novel therapeutic opportunities.

## 1. Introduction

Fatty acids (FAs) are fundamental lipid components that play a central role in several physiological processes within the human body. Beyond their energy-providing function, they have an important structural role in determining the properties of the cell membrane, and they also serve as signaling mediators, influencing cellular pathways at various levels [1]. They influence key blood lipids, such as low-density lipoprotein cholesterol, high-density lipoprotein cholesterol, and triglycerides, all of which play significant roles in the onset and progression of cardiovascular diseases (CVDs) [2], including hypertension.

FAs are mainly classified according to the length and the degree of saturation of their carbon chain. In this study, we focused on plasma long- and very-long-chain FAs, which can be divided into saturated (SFAs), monounsaturated (MUFAs), and polyunsaturated fatty acids (PUFAs). Plasma FA distribution is strongly influenced by dietary intake and can provide useful insights into the body’s metabolic state. For instance, the ratio of ω3 and ω6 PUFAs can be indicative of inflammation levels [3], and specific FA signatures could reflect underlying systemic diseases. Different types of FAs influence cardiovascular health through mechanisms such as lipid metabolism, inflammation, endothelial function, and oxidative stress. As a modifiable risk factor, dietary intake of FAs has long been studied for its potential impact on cardiometabolic health; nevertheless, dietary guidelines for FA intake in the context of CVD prevention remain controversial [4], and further studies are necessary to clarify the impact of the different FA subtypes.

Circulating FA levels have also been associated with hypertensive risk [4,5,6,7], although the findings are not homogeneous due to the different experimental settings and the complex etiology of hypertension. One of the most common forms of secondary hypertension is primary aldosteronism (PA), which is a hormonal disorder characterized by an excessive aldosterone production by the adrenal glands, independent of the renin-angiotensin system and other physiological regulators [8].

PA can be either bilateral, when both the adrenal glands are responsible for the excessive aldosterone secretion, or unilateral. The unilateral disease, mainly due to an aldosterone-producing adenoma, is usually the one with the most severe phenotype. Despite the high estimated prevalence, ranging from 5% in normotensive subjects to 20% in patients with treatment-resistant hypertension, PA is often underdiagnosed, with specific testing typically occurring after the development of serious comorbidities [9,10]. A better understanding of the pathophysiological mechanisms underlying this condition is indeed crucial for an accurate diagnosis and its effective management, since patients with PA are at a significantly greater risk of organ damage and cardiovascular complications compared to those with essential hypertension (EH) [11].

This study aimed to investigate the differences in the plasma FA profiles of hypertensive subjects, focusing on the comparison between patients affected by unilateral PA and patients with a diagnosis of EH. Furthermore, we explored the impact of pharmacological and surgical therapies on the FA distribution of PA subjects, following them in a longitudinal analysis. This work may contribute to a deeper understanding of the intricated relationship between FAs and hypertension, potentially paving the way for an improved diagnostic process and a better comprehension of PA.

## 2. Materials and Methods

### 2.1. Patient Selection

This project included participants referring to the Hypertension Unit of the Verona University Hospital (Verona, Italy), recruited between 2012 and 2019.

Patients were initially screened for PA using the aldosterone-to-renin ratio (ARR), following previously established protocols according to the year of enrollment [12,13]. A diagnosis of PA was confirmed in those with elevated ARR values through the intravenous salt loading test (IV-SLT). The confirmatory test was considered positive if plasma aldosterone levels remained above 50 pg/mL after intravenous saline infusion [14]. In cases where aldosterone levels failed to adequately suppress following the saline load, adrenal venous sampling (AVS) was conducted after a CT scan to determine the subtype of the disease (either bilateral or unilateral primary aldosteronism). AVS was deemed technically successful if the selectivity index—defined as the cortisol ratio between the adrenal vein and the inferior vena cava—exceeded 2. Lateralized aldosterone secretion, indicative of unilateral disease, was defined by a lateralization index greater than 2, based on cortisol-corrected aldosterone ratios between the dominant and non-dominant adrenal veins [15]. Patients who tested positive in both the screening and confirmatory steps were diagnosed with PA and further classified into disease subtypes (either bilateral PA or unilateral PA) based on AVS. Subjects with an ARR value below the screening threshold and those who had a positive screening, but a negative confirmatory test (i.e., IV-SLT) were classified as having EH. Only patients with a diagnosis of unilateral PA on AVS were enrolled in this study.

Demographic characteristics, relevant medical history, and clinical information were gathered at baseline. Blood samples were collected at enrollment, and for some PA patients, also after therapy with mineralocorticoid receptor antagonist (MRA) and/or after adrenalectomy. At the time of blood sampling, subjects were at pharmacological washout from drugs interfering with ARR determination; only verapamil and doxazosin were allowed. Routine laboratory tests, including measurement of biochemical and hormonal parameters, were performed at the laboratory of the Clinical Chemistry Institute of the Verona University Hospital. The study was conducted in accordance with the principles contained in the Declaration of Helsinki. Informed consent was obtained from each patient involved in the study, and the protocol was approved by the local Ethics Committee.

### 2.2. Plasma Sample Collection

The clinical routine blood draw was obtained through venipuncture, collected in Vacutainer (BD, Franklin Lakes, NJ, USA) citrate collection tubes, and centrifuged at 1500× *g* for 15 min at 4 °C. Plasma was collected and stored at −20 °C.

### 2.3. Fatty Acid Extraction

Plasma long- and very-long-chain FAs were extracted with organic solvents and analyzed by gas chromatography according to a protocol adapted from the work of Masood and colleagues [16] and applied in several studies by our group on patients with coronary artery disease (CAD) [17,18]. Each FA calibration standard was purchased from Sigma-Aldrich and dissolved in a chloroform–isopropanol solution (7:11 *v*/*v*). Standards concentrations used for the analysis are shown in Table 1. Heptadecanoic acid methyl ester (C17:0, Sigma-Aldrich, Burlington, MA, USA) was used as an internal standard and dissolved in the chloroform–isopropanol solution to a final concentration of 10 mg/mL. FAs were extracted starting from 100 μL of plasma citrate and 100 μL of calibration standard, to which were added 10 μL of internal standard. The transesterification reaction was carried out by adding 2 mL of a methanol/acetyl chloride solution (99:1 *v*/*v*) containing 0.1 g/L of butylated hydroxytoluene and incubating at 100 °C for 1 h. After the transesterification, samples were cooled to room temperature. To isolate fatty acid methyl esters (FAME) through liquid–liquid extraction, 1 mL of water and 1 mL of hexane were added to the vials. The samples were vortexed for 30 s and centrifuged at 1300× *g* for 2 min. The organic phase was collected and placed in a new vial. Extraction was performed twice. To increase sensitivity, FAMEs were dried and then reconstituted in 0.5 mL and 1 mL of hexane for samples and calibration standards, respectively. FA extraction was successfully performed in 22 samples of patients with PA, collected for the screening, in a group of 60 EH patients matched for age and sex, and in 10 PA patients at follow-up.

### 2.4. Statistical Analysis

Data analysis was carried out with Python 3.10.12, using the following libraries: pandas 2.2.2 and numpy 1.26.4 for data processing; scipy 1.13.1 and statsmodels 0.14.4 for statistical tests; matplotlib 3.8.0 and seaborn 0.13.2 for data visualization. Shapiro–Wilk test was used to assess the normality of the variables. Descriptive statistics were calculated to summarize the main characteristics of the variables. Mean and standard deviation (SD) were used for normally distributed variables, while median and interquartile range (IQR) were used for non-normally distributed ones. For the comparison between PA and EH, *t*-test and Mann–Whitney U test were applied for normally distributed variables and non-normally distributed variables, respectively. The chi-squared test was used to assess differences between categorical variables. For the longitudinal analysis, the Friedman test was used to compare the differences between the three time points, while the Wilcoxon-Mann–Whitney test was used for pairwise comparisons. The level of significance was set at α = 0.05. Given the exploratory nature of this pilot study, no formal adjustment for multiple comparisons was applied.

## 3. Results

### 3.1. Comparison Between PA and EH Patients

Clinical and biochemical characteristics of the assessed cohort are reported in Table 2. The mean age was 49 ± 12 years, and 86.6% were males. The 22 subjects with unilateral PA presented the typical biochemical hallmarks of the disease. Compared to EH (*n* = 60), they had significantly higher aldosterone (median [IQR]: PA = 263.0 [182.0, 364.0] pg/mL vs. EH = 157.5 [121.75, 197.5] pg/mL, *p* < 0.001, 67% higher in PA) and lower renin (median [IQR]; PA = 4.38 [2.0, 5.52] pg/mL vs. EH = 7.89 [4.11, 12.68] pg/mL, *p* = 0.01, 80.1% lower in PA) levels, and a markedly higher aldosterone to renin ratio (median [IQR]: PA = 60.0 [38.89, 90.5] vs. EH = 23.69 [10.79, 35.18], *p* < 0.001, 153.3% higher in PA). Moreover, they showed a lower serum potassium concentration (mean ± SD: PA = 3.46 ± 0.66 mmol/L vs. EH = 3.89 ± 0.3 mmol/L, *p* < 0.001, 11% lower in PA), which is a common feature among patients affected by PA, and a lower serum chloride concentration (mean ± SD: PA = 102.19 ± 2.83 mmol/L vs. EH = 103.6 ± 2.37 mmol/L, *p* = 0.05, 1.36% lower in PA). Dyslipidemia was less prevalent in patients with PA than in those with EH (PA = 35.00% vs. EH = 52.63%, *p* < 0.05), while hypertensive cardiopathy was more frequent in PA (PA = 83.33% vs. EH = 56.0%, *p* < 0.0001). No differences were found between PA and EH patients in the prevalence of diabetes and smoking habits. Additionally, none of the subjects had a documented history of CAD, and fewer than 10% were on statin therapy at enrollment.

**Figure 1:**
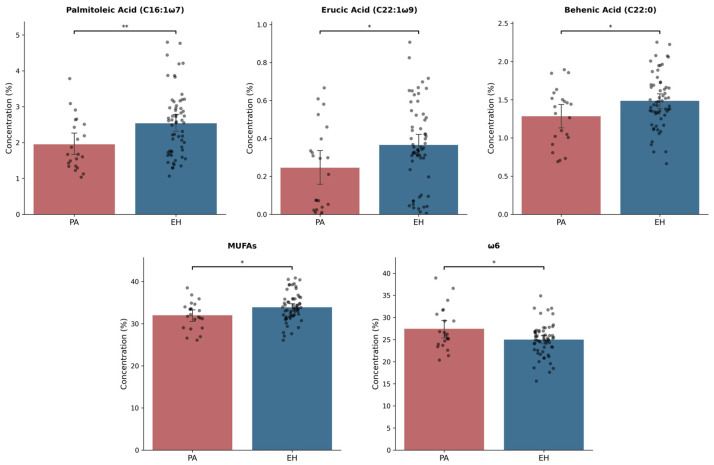
Significantly different plasma FA levels between PA (in red) and EH (in blue) patients. FA plasma concentration is expressed as a percentage of total FAMEs. Differences across time points were assessed using either a *t*-test or Mann–Whitney U test, according to the normality of the variable. MUFAs, monounsaturated fatty acids; ω6, omega-6 polyunsaturated fatty acids. Significance levels: * *p* < 0.05, ** *p* < 0.005.

Comparing the FA profiles (Table 3), PA displayed lower plasma levels of behenic acid (mean ± SD: PA = 1.28 ± 0.38% vs. EH = 1.48 ± 0.35%, *p* = 0.03, 13.5% lower in PA) and monounsaturated fatty acids (MUFAs) (mean ± SD: PA = 32.0 ± 3.3% vs. EH = 33.92 ± 3.38%, *p* = 0.02, 5.6% lower in PA) than EH. Among MUFAs, palmitoleic acid (median [IQR]: PA = 1.67 [1.37, 2.49] % vs. EH = 2.57 [1.75, 2.98] %, *p* = 0.005, 35% lower in PA) and erucic acid (median [IQR]: PA = 0.25 [0.04, 0.38] % vs. EH = 0.35 [0.27, 0.51] %, *p* = 0.02, 28.6% lower in PA) concentrations were significantly lower in PA. PA patients also showed higher levels of ω6 polyunsaturated fatty acids (PUFAs) compared to EH (mean ± SD: PA = 27.44 ± 4.84% vs. EH = 25.0 ± 3.64%, *p* = 0.02, 9.8% higher in PA) (Figure 1).

### 3.2. Effects of Pharmacological and Surgical Therapies on the FA Profile of PA Patients

To test whether the pharmacological and surgical treatments could alter the plasma FA distribution in PA patients, a longitudinal study was performed in just 10 male subjects, due to sample availability. Plasma samples were collected at three distinct time points: at enrollment (T0), when subjects were not receiving any antihypertensive medications; on the day of surgery (T1), when patients were under MRA treatment; and six months after adrenalectomy (T2). Significant changes were observed in several clinical and biochemical parameters after treatment, as illustrated in Table 4. As expected, therapy led to a 46.3% reduction in aldosterone (median [IQR]: T0 = 247.5 [194.0, 301.5] pg/mL vs. T2 = 133.00 [94.25, 154.50] pg/mL, *p* = 0.02) and a rise in renin levels (median [IQR]: T0 = 2.00 [1.62, 4.80] pg/mL vs. 10.44 [3.93, 11.95] pg/mL, *p* = 0.05), with a consequent reduction in the ARR (median [IQR]: T0 = 90.5 [41.55, 187.0] vs. T2 = 12.46 [9.47, 29.76], *p* = 0.04). Systolic and diastolic blood pressure decreased progressively from enrollment through MRA treatment to adrenalectomy, with statistically significant reductions from T0 to T2 (median [IQR]: T0 = 160 [150, 170]/100 [90, 100] mmHg vs. T2 = 132.50 [125, 138.75]/82.50 [80, 88.75] mmHg, *p* = 0.004/0.016). Serum potassium levels increased significantly over time, rising from 2.92 [2.76, 3.56] mmol/L at T0 to 4.33 [4.06, 4.53] mmol/L at T2 (*p* = 0.002), reflecting correction of hypokalemia. Other relevant clinical parameters, including eGFR, serum sodium, and serum chloride concentration, did not demonstrate significant changes across the treatment period.

**Figure 2:**
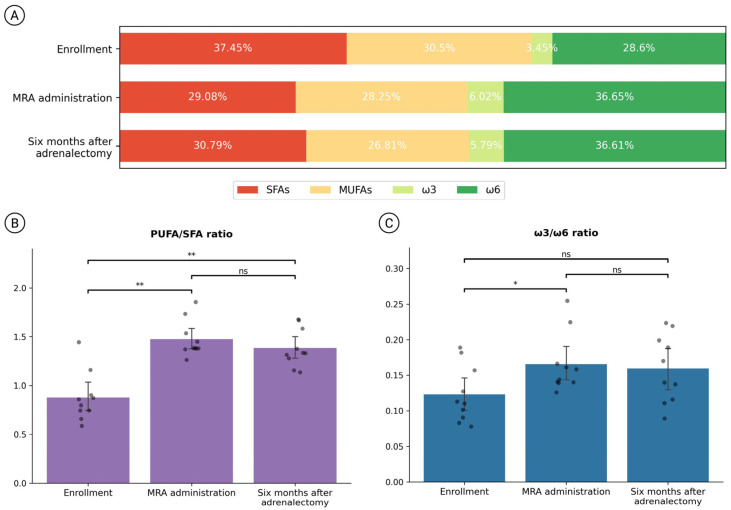
FA profile comparisons from baseline (pharmacological washout) throughout the follow-up period (MR antagonist administration and 6 months after surgery): (**A**) stacked bar chart showing the changes in the distribution of the major FA classes over time; (**B**) bar chart representing the PUFA/SFA proportion in the three experimental stages; (**C**) bar chart representing the ω3/ω6 proportion in the three experimental sampling times. SFAs, saturated fatty acids; MUFAs, monounsaturated fatty acids; PUFAs, polyunsaturated fatty acids. Significance levels: ns *p* ≥ 0.05, * *p* < 0.05, ** *p* < 0.005.

Interestingly, PA patients exhibited a substantial change in their FA profile after the pharmacological treatment (Table 4). For most FA species, this shift in the distribution was also preserved after adrenalectomy (Figure 2A). Following MRA administration, PA patients displayed a decrease in total saturated fatty acid (SFA) plasma concentration (median [IQR]: T0 = 38.92 [37.28, 39.5] % vs. T1 = 28.94 [28.73, 30.14] %, *p* = 0.004, 25.6% decrease), and an increase in PUFA levels (median [IQR]: T0 = 31.69 [28.48, 34.6] % vs. T1 = 41.32 [39.92, 44.01] %, *p* = 0.006, 30.4% increase), especially as regards ω3 PUFAs which were almost doubled compared to the basal levels (median [IQR]: T0 = 3.25 [2.64, 4.28] % vs. T1 = 5.7 [5.29, 6.14] %, *p* = 0.004, 75.4% increase).

Consistent with the changes induced by the pharmacological intervention, total PUFA levels were higher than those at enrollment, as well as six months after the adrenalectomy (median [IQR]: T0 = 31.69 [28.48, 34.6] % vs. T2 = 41.53 [39.78, 44.85] %, *p* = 0.004, 31% increase). In addition, surgery led to a 7.5% increase in SFAs (median [IQR]: T1 = 28.94 [28.73, 30.14] % vs. T2 = 31.1 [29.64, 31.32] %, *p* = 0.002) and a slight reduction in total MUFAs (median [IQR]: T1 = 28.69 [26.55, 30.86] % vs. T2 = 26.82 [25.66, 27.75] %, *p* = 0.03, 6.5% decrease) compared to the MRA treatment.

Considering the proportion between FA classes, the PUFA/SFA ratio markedly rose after MRA administration, with a modest decrease after adrenalectomy (Figure 2B). Within the PUFA category, a significant increase in the ω3/ω6 ratio was observed at T1, with its value remaining nearly unchanged at T2 (Figure 2C).

## 4. Discussion

The aim of this study was to better characterize the pathophysiological mechanisms underlying PA through the analysis of plasma long- and very-long-chain FAs, focusing on the comparison with EH subjects and on the modifications induced by the pharmacological and surgical treatments in PA.

### 4.1. Differences in Plasma FA Distribution Between PA and EH

FA profiles analysis in the comparison between PA and EH patients revealed that PA patients had significantly lower levels of monounsaturated fatty acids (MUFAs), particularly regarding palmitoleic and erucic acids. Palmitoleic acid is a ω7 MUFA that could modulate several metabolic responses acting as a lipokine, including increased insulin sensitivity in muscles, β cell proliferation, prevention of endoplasmic reticulum stress, and lipogenic activity in white adipocytes [19]. This MUFA has shown various beneficial effects in both animal and cell studies, but its positive impact on humans is still debated [20]. Even though the role of palmitoleic acid in hypertension remains largely unknown and even controversial, in rat models, it was found that circulating palmitoleic acid was inversely associated with the risk of primary hypertension, with a protective role against hypertension, potentially improving aortic remodeling through the inhibition of NF-kB-mediated inflammation [21]. Moreover, the other significantly varying MUFA was the ω9 erucic acid, which can be primarily found in some vegetable oils such as canola oil. The existing studies regarding its effects on human health are limited and often controversial, but, in recent years, its role has been completely reconsidered. Although very high doses of this FA may lead to cardiac lipidosis in mice, reasonable amounts of erucic acid have demonstrated abundant anti-inflammatory and antioxidant properties both in vitro and in vivo [22,23]. Moreover, PA patients showed lower plasma levels of behenic acid, which is a very-long-chain saturated fatty acid (SFA). Unlike other SFAs, whose intake should be reduced due to their deleterious effects [24], very-long-chain SFAs have shown potential health benefits, especially on heart disease, diabetes, mortality, and aging [25]. Higher circulating levels of behenic and lignoceric acids have been linked to a lower risk of cardiovascular disease, all-cause mortality, and unhealthy aging events [26,27]. Therefore, lower plasma concentrations of palmitoleic, erucic, and behenic acids could reflect a negative condition due to a more prominent inflammatory state in PA subjects, potentially contributing to the higher risk of organ damage typical of PA.

In addition to lower MUFAs, PA patients also exhibited significantly higher plasma concentration of ω6 polyunsaturated fatty acids (PUFAs) than EH, which might contribute to the higher inflammatory milieu. This difference may be related to aldosterone, which is oversecreted in PA. Studies on amphibian epithelial cells and rat colonocytes agreed on demonstrating that long-term exposure to aldosterone causes a remodeling of the FA content in phospholipids, favoring ω6 PUFAs over MUFAs [28,29]. These findings are consistent with another work showing that aldosterone treatment reduced MUFA and raised PUFA levels in intestinal epithelial cell phospholipids [30]. In general, previous studies suggested a complex interplay between aldosterone and FAs, with reciprocal influences. For instance, certain non-esterified FAs modulated aldosterone production in human adrenocortical HAC15 cells, stimulating the expression of *CYP11B2* [31], while aldosterone-induced alteration of FA metabolism was required for epithelial Na^+^ transport in toad urinary bladder [28,32].

Taken together, our findings interpreted on the basis of previous literature data suggest that the differences in the FA distribution in PA patients are likely secondary to aldosterone excess and contribute to the higher inflammatory state in comparison with EH.

### 4.2. Improvement of the FA Profile in PA Patients After Treatment

The findings presented in this study reveal that both pharmacological and surgical interventions could significantly alter the plasma FA profile in patients with PA. These changes were particularly evident in relation to the shifts observed in the distribution of SFAs and PUFAs, especially those belonging to the ω3 PUFA family. The administration of MRAs led to a notable decrease in total SFA levels and a concurrent increase in PUFA abundance, with a marked upregulation of ω3 PUFAs. These changes were maintained even after adrenalectomy, suggesting that either the MRA-induced modifications in FA metabolism could persist beyond the surgical procedure, or both interventions affected the same biological process, acting synergistically. To our knowledge, the exact molecular mechanisms underlying these significant variations in the FA profile have never been investigated. Therefore, we can speculate that both initial MRA administration and subsequent lower circulating aldosterone levels due to the removal of the source of its production by adrenalectomy could reduce mineralocorticoid receptor activation. It is well established that a lower mineralocorticoid receptor activity can downregulate genes involved in inflammation and fibrosis [33]; therefore, a lower inflammatory state could be reflected by the different plasma FA distribution. Alternatively, the mineralocorticoid receptor might directly affect the expression of genes that regulate FA metabolism. For instance, MRA administration was associated with a lower expression of FA transporters in epicardial fat, indicating that MRAs may have a protective effect on heart remodeling by reducing the accumulation of FAs [34]. Nevertheless, the relation between mineralocorticoid receptor activity and FA metabolism remains largely unexplored, certainly requiring further investigation.

Another aspect that is worth discussing is the dichotomous role of arachidonic acid (AA), the most abundant ω6 PUFA in our body that acts as a precursor for several signaling molecules such as prostaglandins, thromboxanes, leukotrienes, and lipoxins [35]. In this work, we found that PA displayed a higher plasma concentration of ω6 PUFAs compared to EH, even though individual levels of AA and its precursor, linoleic acid, were higher but not significantly different between groups. Consistent with this result, higher circulating AA levels were recently observed by Yang and colleagues in subjects with a classical form of unilateral PA compared to patients with a different PA subtype, as well as in aldosterone-producing adenomas compared to adjacent healthy tissues. In addition, they showed how AA could stimulate *CYP11B2* expression in vitro, leading to oxidative stress and apoptosis in adrenocortical cells [36]. On the other hand, our longitudinal analysis revealed that AA abundance increased following pharmacological treatment, and this elevation persisted after adrenalectomy. This could be counterintuitive since elevated levels of AA appeared to be a peculiar metabolic characteristic of PA. However, it is well established that certain downstream products of AA metabolism play an important role in several physiological responses, including the resolution of inflammation, hemostasis, and blood pressure regulation. In an observational study by Luther and colleagues, PA patients displayed, after treatment with either MRAs or adrenalectomy, higher circulating levels of epoxyeicosatrienoic acids (EETs), a class of signaling molecules derived from AA with vasodilatory and anti-inflammatory properties. These findings were also supported by data from mouse models treated with aldosterone, which showed reduced EET levels and an increased conversion into less active metabolites [37]. A downregulation of 14,15-epoxyeicosatrienoic acid, an EET that has a beneficial effect in vascular remodeling, was also associated with abdominal aortic calcification in subjects with PA [38]. However, it is important to mention that another work showed how aldosterone did not affect or stimulate the release of endothelial EETs, and no differences were detected in eicosanoid plasma concentrations between PA and EH [39]. We can therefore speculate that the post-treatment rise in AA and total ω6 levels observed in PA patients may lead to a higher production of beneficial byproducts, including EETs. However, this hypothesis is not yet supported by experimental data and requires additional investigation to establish the potential relationship between AA and EET in PA.

Similarly, the observed increase in the PUFA/SFA ratio after MRA treatment and its subsequent preservation post-adrenalectomy are of particular interest. This shift towards a more favorable lipid profile is associated with several health benefits, including a reduced risk of CVD and improved metabolic health [40]. The increase in the ω3/ω6 PUFA ratio further supports the notion that these treatments promote a healthier lipid profile [41]. A practical way to actively improve the FA profile is the modulation of the dietary FA intake. A healthy nutritional regimen, such as that of the Mediterranean diet, is low in saturated and trans FAs and rich in MUFAs and PUFAs. Such dietary characteristics in terms of FA content are associated with a reduced incidence of several cardiometabolic risk factors, including hypertension and inflammation [42,43]. Nevertheless, the relationship between dietary FA intake, plasma FA levels, and cardiovascular health has been a source of significant debate over the past few decades [44]. This ongoing controversy is fueled by the complexity of dietary patterns, which makes it difficult to isolate the contributions of individual FAs using non-standardized methods for the analysis of FAs (and the different biological sources of FAs investigated), and by the lack of dedicated controlled trials investigating the relation between specific FA types and metabolic health. In addition, a large-scale epidemiological investigation, recently published, revealed moderate correlations between measured plasma phospholipid FAs and estimated dietary FA intake: SFAs (r = 0.11), MUFAs (r = 0.27), and PUFAs (r = 0.28). Nevertheless, these authors emphasized that the health risks linked to SFAs differ significantly across their subtypes, challenging current broad dietary guidelines that focus only on reducing total SFA intake. Their results suggested a beneficial role for ω3 PUFAs and linoleic acid in cardiovascular health, in line with our observation [45]. In conclusion, this longitudinal analysis provides compelling evidence that both pharmacological and surgical interventions can not only significantly impact the expected clinical parameters (i.e., ARR, blood pressure, and potassium levels) but also promote a favorable shift in the plasma FA distribution in PA patients. The observed shifts towards a less inflammatory lipid profile underscore the effective benefits of these interventions on the management of PA-related metabolic complications. Nevertheless, further research is needed to better elucidate and confirm, at the molecular level, the underlying mechanisms and to assess the long-term clinical implications of these findings.

### 4.3. Study Limitations and Future Perspectives

This study highlighted the presence of specific plasma FA alterations connected to PA that may have relevant clinical implications, including the use of FAs as potential new markers for risk stratification of this condition. Furthermore, considering that recently higher dietary MUFA intake was found to be protective against hypertension [46], a suggestive hypothesis is that this FA supplementation may become a promising complementary approach to the conventional therapeutic strategies for PA management. The PA population investigated in our study was carefully characterized at baseline and at follow-up, allowing for the longitudinal observation of the therapy-induced FA profile modifications in the same patient. However, several limitations of this study must be considered. Firstly, the relatively small sample size may have limited the statistical power for the detection of significant differences, and it may have also hampered the generalizability of the longitudinal study. Secondly, the absence of a control group of normotensive individuals limits the possibility of generalizing the conclusions of this work, even if the scope of our study was to highlight the peculiarities of the FA profile of PA patients in the frame of hypertension. A normotensive control group would have helped to disentangle PA-specific signatures from those related merely to hypertension. In addition, another potential confounding factor was the lack of a standardized patient diet, as plasma FA levels are determined by both dietary intake and endogenous metabolic processes [47]. A fixed dietary regimen would have attenuated the dietary impact on FA levels, allowing us to focus exclusively on the metabolic aspects of FA handling in PA, while uncontrolled or unknown dietary regimens in terms of FA intake can potentially give rise to misleading results. However, the precise association between diet and circulating FA levels is still a matter of debate, particularly for specific classes of FAs [48]. Future studies should include larger and diversified cohorts, as well as controlled diets or dietary questionnaires, to address these limitations and confirm our hypotheses. Dietary recommendations should reflect the latest evidence from studies on circulating FAs and randomized controlled trials.

Moreover, our study lacks the validation of our observations through in vitro and in vivo approaches. For instance, further experiments should focus on exploring cellular pathways linking aldosterone excess to FA metabolism, potentially identifying direct effects of mineralocorticoid receptor signaling on FA regulatory genes.

Finally, our work, in line with other research, indicates that FAs are emerging as promising biomarkers for hypertension and PA, particularly for early detection and risk assessment. Modulating FA metabolism or specific dietary supplementation might also offer novel therapeutic avenues for managing these conditions. Further research is needed to fully elucidate the role of FAs in hypertension, the possible beneficial role of dietary approaches, and the translation of these findings into clinical practice.

## 5. Conclusions

This pilot study revealed that patients with PA, compared to those with EH, exhibit a distinct plasma FA profile, characterized by lower levels of MUFAs and behenic acid, and higher levels of ω6 PUFAs, suggesting a more pronounced inflammatory state in PA. Crucially, both MRA therapy and adrenalectomy positively shifted the FA profile in PA patients, decreasing total SFAs and increasing total PUFAs, particularly ω3 PUFAs. Our study addresses the usefulness of plasma FA profiling for a better understanding of the molecular mechanisms involved in the pathophysiology of PA. This finding also underscores the role of FA as markers for PA characterization and risk stratification, suggesting new potential therapeutic avenues, besides traditional approaches, for the management of this condition. These future perspectives, including the hypothesis of dietary MUFA supplementation, will need further confirmatory studies such as dedicated clinical trials.

## Figures and Tables

**Table 1:** Composition of the FA calibration standard mix.

PubChem Compound ID	Common Name	Omega Nomenclature	Concentration (mg/mL)
3893	Lauric acid	C12:0	0.25
11005	Myristic acid	C14:0	0.25
985	Palmitic acid	C16:0	0.25
5281	Stearic acid	C18:0	0.25
10467	Arachidic acid	C20:0	0.25
8251	Behenic acid	C22:0	0.10
11197	Lignoceric acid	C24:0	0.10
10469	Cerotic acid	C26:0	0.10
445638	Palmitoleic acid	C16:1(*n*-7)	0.25
445639	Oleic acid	C18:1(*n*-9)	0.25
5282768	Eicosenoic acid	C20:1(*n*-9)	0.25
5281116	Erucic acid	C22:1(*n*-9)	0.25
5280934	α-Linolenic acid	C18:3(*n*-3)	0.10
5312508	Stearidonic acid	C18:4(*n*-3)	0.10
446284	Eicosapentaenoic acid	C20:5(*n*-3)	0.09
445580	Docosahexaenoic acid	C22:6(*n*-3)	0.10
5280450	Linoleic acid	C18:2(*n*-6)	0.25
6439848	Eicosadienoic acid	C20:2(*n*-6)	0.25
444899	Arachidonic acid	C20:4(*n*-6)	0.25

**Table 2:** Summary of the clinical and biochemical characteristics of the cohort studied.

	PA (*n* = 22)	EH (*n* = 60)	*p*
Age *	49 ± 10	48 ± 13	0.809
Sex (Males %) ^X^	86.36	86.67	1.000
BMI (kg/m2) ^#^	28.0 [24.5, 30.5]	27.0 [24.57, 31.0]	0.952
Dyslipidemia (%) ^X^	35.00	52.63	0.018
Hypertensive cardiopathy (%) ^X^	83.33	56.90	<0.001
Diabetes (%) ^X^	4.55	1.72	0.460
Smoking (%) ^X^	45.00	44.64	1.000
Systolic BP (mmHg) ^#^	147.5 [133.75, 170.0]	147.5 [140.0, 160.0]	0.913
Diastolic BP (mmHg) ^#^	90.0 [80.0, 100.0]	95.0 [85.0, 100.0]	0.659
Glycemia (mmol/L) ^#^	4.8 [4.59, 5.02]	4.8 [4.6, 5.2]	0.700
Total Cholesterol (mmol/L) *	5.07 ± 0.88	5.22 ± 0.93	0.552
LDL (mmol/L) *	3.05 ± 0.78	3.33 ± 0.86	0.284
HDL (mmol/L) ^#^	1.42 [1.14, 1.5]	1.38 [1.1, 1.59]	0.868
Triglycerides (mmol/L) ^#^	0.96 [0.79, 1.36]	1.07 [0.75, 1.39]	0.715
Aldosterone (pg/mL) ^#^	263.0 [182.0, 364.0]	157.5 [121.75, 197.5]	<0.001
Renin (pg/mL) ^#^	4.38 [2.0, 5.52]	7.89 [4.11, 12.68]	0.011
ARR ^#^	60.0 [38.89, 90.5]	23.69 [10.79, 35.18]	<0.001
Serum Na+ (mmol/L) *	142.53 ± 2.4	141.25 ± 2.1	0.038
Serum K+ (mmol/L) *	3.46 ± 0.66	3.89 ± 0.3	<0.001
Serum Cl− (mmol/L) *	102.19 ± 2.83	103.6 ± 2.37	0.050
eGFR (mL/min/1.73 m2) *	78.1 ± 17.81	83.81 ± 17.55	0.245

* Variable normally distributed in both groups. It is presented as mean ± SD, and *t*-test was applied. ^X^ Categorical variable; chi-square test was applied. ^#^ Variable not normally distributed in at least one group. It is presented as median [IQR], and Mann–Whitney U test was applied.

**Table 3:** Differences in the FA profile between PA and EH.

	PA (*n* = 22)	EH (*n* = 60)	*p*
SFAs ^#^	38.51 [36.71, 39.5]	38.06 [36.47, 39.78]	0.971
Lauric Acid—C12:0 ^#^	0.13 [0.08, 0.2]	0.11 [0.08, 0.23]	0.913
Myristic Acid—C14:0 ^#^	1.66 [1.08, 2.19]	1.45 [1.18, 1.86]	0.794
Palmitic Acid—C16:0 *	24.25 ± 2.18	24.86 ± 1.45	0.146
Stearic Acid—C18:0 *	9.0 ± 1.09	8.83 ± 0.97	0.497
Arachidic Acid—C20:0 *	0.36 ± 0.06	0.39 ± 0.08	0.124
Behenic Acid—C22:0 *	1.28 ± 0.38	1.48 ± 0.35	0.029
Lignoceric Acid—C24:0 *	0.8 ± 0.18	0.88 ± 0.22	0.129
Cerotic Acid—C26:0 ^#^	0.07 [0.06, 0.1]	0.08 [0.07, 0.1]	0.211
UFAs ^#^	61.49 [60.5, 63.29]	61.94 [60.22, 63.53]	0.971
MUFAs *	32.0 ± 3.3	33.92 ± 3.38	0.025
Palmitoleic Acid—C16:1(*n*-7) ^#^	1.67 [1.37, 2.49]	2.57 [1.75, 2.98]	0.005
Oleic Acid—C18:1(*n*-9) *	29.55 ± 2.82	30.76 ± 3.08	0.112
Eicosenoic Acid—C20:1(*n*-9) ^#^	0.23 [0.18, 0.29]	0.24 [0.21, 0.3]	0.423
Erucic Acid—C22:1(*n*-9) ^#^	0.25 [0.04, 0.38]	0.35 [0.27, 0.51]	0.023
PUFAs ^#^	28.94 [26.71, 33.8]	28.23 [25.53, 29.69]	0.139
ω3 ^#^	2.65 [2.34, 3.86]	2.62 [2.23, 3.18]	0.333
α-Linolenic Acid—C18:3(*n*-3) ^#^	0.26 [0.21, 0.33]	0.21 [0.18, 0.29]	0.060
Stearidonic Acid—C18:4(*n*-3) ^#^	0.21 [0.09, 0.29]	0.24 [0.21, 0.29]	0.110
Eicosapentaenoic Acid—C20:5(*n*-3) ^#^	0.26 [0.2, 0.53]	0.21 [0.16, 0.31]	0.093
Docosahexaenoic Acid—C22:6(*n*-3) *	2.2 ± 0.79	2.02 ± 0.6	0.287
ω6 *	27.44 ± 4.84	25.0 ± 3.64	0.971
Linoleic Acid—C18:2(*n*-6) *	22.16 ± 2.72	20.95 ± 2.85	0.913
Eicosadienoic Acid—C20:2(*n*-6) *	0.23 ± 0.05	0.21 ± 0.04	0.794
Arachidonic Acid—C20:4(*n*-6) ^#^	4.27 [2.9, 5.87]	3.81 [2.45, 4.67]	0.146
ω3/ω6 Ratio ^#^	0.1 [0.09, 0.13]	0.11 [0.09, 0.13]	0.497
PUFA/SFA Ratio ^#^	0.75 [0.68, 0.87]	0.74 [0.66, 0.81]	0.124

FA plasma levels are expressed as a percentage of total FAMEs. SFAs, saturated fatty acids; UFAs, unsaturated fatty acids; MUFAs, monounsaturated fatty acids; PUFAs, polyunsaturated fatty acids. * Variable normally distributed in both groups. It is presented as mean ± SD, and *t*-test was applied. ^#^ Variable not normally distributed in at least one group. It is presented as median [IQR], and Mann–Whitney U test was applied.

**Table 4:** Therapy-related variations of the clinical parameters and FA profile of 10 PA patients first after pharmacological treatment (T1) and then after surgical procedure (T2).

	Enrollment (T0)	MRA Treatment (T1)	Adrenalectomy (T2)	Overall *p*	T0–T1 *p*	T1–T2 *p*	T0–T2 *p*
Aldosterone (pg/mL)	247.5 [194.0, 301.5]	234.5 [220.25, 427.75]	133.00 [94.25, 154.50]	0.420	0.500	0.375	0.020
Renin (pg/mL)	2.00 [1.62, 4.80]	2.00 [1.73, 2.00]	10.44 [3.93, 11.95]	0.050	1.000	0.125	0.055
ARR	90.5 [41.55, 187.0]	104.25 [68.12, 117.88]	12.46 [9.47, 29.76]	0.280	1.000	0.219	0.039
Systolic BP (mmHg)	160 [150, 170]	140 [135, 142.50]	132.50 [125, 138.75]	0.008	0.063	0.063	0.004
Diastolic BP (mmHg)	100 [90, 100]	90 [87.50, 95]	82.50 [80, 88.75]	0.014	0.250	0.063	0.016
eGFR (mL/min/1.73 m2)	79.10 [64.96, 88.95]	76.21 [67.58, 88.81]	69.82 [59.20, 83.89]	0.264	1.000	0.084	0.098
Serum Na (mmol/L)	142 [141, 143]	140.50 [139, 143.25]	140 [140, 143.75]	0.093	0.188	1.000	0.129
Serum K (mmol/L)	2.92 [2.76, 3.56]	3.60 [3.26, 3.96]	4.33 [4.06, 4.53]	0.003	0.313	0.055	0.002
Serum Cl (mmol/L)	103 [99, 103]	103 [101, 104]	102.50 [101, 103.75]	0.441	1.000	0.375	0.781
SFAs	38.92 [37.28, 39.5]	28.94 [28.73, 30.14]	31.1 [29.64, 31.32]	0.001	0.004	0.002	0.004
Lauric Acid (C12:0)	0.11 [0.08, 0.19]	0.07 [0.06, 0.11]	0.16 [0.08, 0.3]	0.020	0.065	0.006	0.375
Myristic Acid (C14:0)	1.39 [0.89, 1.99]	0.95 [0.73, 1.33]	1.68 [1.39, 2.07]	0.020	0.084	0.002	0.770
Palmitic Acid (C16:0)	23.91 [23.56, 25.51]	19.81 [19.29, 20.15]	20.04 [19.1, 20.63]	0.007	0.004	0.432	0.004
Stearic Acid (C18:0)	8.99 [8.6, 9.55]	6.67 [6.31, 7.14]	7.27 [7.0, 7.89]	0.001	0.002	0.002	0.004
Arachidic Acid (C20:0)	0.39 [0.35, 0.42]	0.3 [0.29, 0.34]	0.31 [0.29, 0.32]	0.061	0.027	0.846	0.004
Behenic Acid (C22:0)	1.43 [1.28, 1.6]	0.73 [0.68, 0.93]	0.72 [0.66, 0.95]	0.061	0.002	0.922	0.004
Lignoceric Acid (C24:0)	0.84 [0.73, 0.94]	0.55 [0.51, 0.74]	0.59 [0.51, 0.71]	0.045	0.006	0.432	0.004
Cerotic Acid (C26:0)	0.07 [0.06, 0.08]	0.01 [0.01, 0.01]	0.01 [0.01, 0.01]	0.001	0.002	0.322	0.002
UFAs	61.08 [60.5, 62.72]	71.06 [69.86, 71.27]	68.9 [68.68, 70.36]	0.001	0.004	0.002	0.004
MUFAs	31.11 [27.44, 33.02]	28.69 [26.55, 30.86]	26.82 [25.66, 27.75]	0.045	0.160	0.027	0.020
Palmitoleic Acid—C16:1(*n*-7)	1.52 [1.37, 1.66]	1.49 [1.13, 1.75]	1.78 [1.24, 2.07]	0.905	0.375	0.322	1.000
Oleic Acid—C18:1(*n*-9)	28.69 [25.62, 30.28]	26.93 [25.39, 28.58]	24.67 [23.76, 25.55]	0.027	0.193	0.020	0.020
Eicosenoic Acid—C20:1(*n*-9)	0.2 [0.18, 0.24]	0.17 [0.17, 0.24]	0.19 [0.16, 0.22]	0.741	0.846	0.492	0.432
Erucic Acid—C22:1(*n*-9)	0.18 [0.03, 0.42]	0.01 [0.01, 0.02]	0.01 [0.01, 0.01]	0.006	0.006	0.193	0.004
PUFAs	31.69 [28.48, 34.6]	41.32 [39.92, 44.01]	41.53 [39.78, 44.85]	0.001	0.006	0.432	0.004
ω3	3.25 [2.64, 4.28]	5.7 [5.29, 6.14]	5.66 [4.35, 7.16]	0.001	0.004	0.770	0.010
α-Linolenic Acid—C18:3(*n*-3)	0.25 [0.21, 0.32]	0.57 [0.45, 0.69]	0.56 [0.43, 0.62]	0.020	0.002	0.695	0.006
Stearidonic Acid—C18:4(*n*-3)	0.23 [0.1, 0.29]	0.06 [0.05, 0.07]	0.06 [0.05, 0.07]	0.061	0.014	0.322	0.014
Eicosapentaenoic Acid—C20:5(*n*-3)	0.32 [0.22, 0.73]	0.79 [0.67, 1.05]	0.98 [0.63, 1.41]	0.067	0.065	0.770	0.027
Docosahexaenoic Acid—C22:6(*n*-3)	2.39 [2.0, 2.96]	4.25 [3.89, 4.71]	3.83 [3.16, 4.85]	<0.001	0.002	0.375	0.010
ω6	29.23 [24.87, 31.44]	35.22 [34.6, 37.16]	35.69 [34.3, 37.31]	0.007	0.006	0.625	0.010
Linoleic Acid—C18:2(*n*-6)	23.1 [20.59, 25.47]	24.62 [23.64, 25.88]	25.55 [23.01, 26.38]	0.150	0.049	0.922	0.084
Eicosadienoic Acid—C20:2(*n*-6)	0.23 [0.21, 0.25]	0.23 [0.2, 0.29]	0.28 [0.25, 0.29]	0.082	0.557	0.492	0.020
Arachidonic Acid—C20:4(*n*-6)	4.87 [3.23, 6.9]	10.75 [9.03, 12.72]	9.97 [8.68, 13.3]	0.008	0.006	0.695	0.004
ω3/ω6 Ratio	0.11 [0.09, 0.15]	0.15 [0.14, 0.17]	0.16 [0.12, 0.2]	0.020	0.049	0.770	0.084
PUFA/SFA Ratio	0.83 [0.75, 0.89]	1.38 [1.38, 1.51]	1.33 [1.29, 1.53]	0.001	0.004	0.049	0.004

FA plasma levels were expressed as a percentage of total FAMEs. Differences across time points were assessed using the Friedman test. Pairwise comparisons between time points were conducted using the Wilcoxon-Mann–Whitney test. SFAs, saturated fatty acids; UFAs, unsaturated fatty acids; MUFAs, monounsaturated fatty acids; PUFAs, polyunsaturated fatty acids.

## Data Availability

The original contributions presented in this study are included in the article. Further inquiries can be directed to the corresponding author.

## Abbreviations

The following abbreviations are used in this manuscript:

AA	Arachidonic Acid
ARR	Aldosterone-to-Renin Ratio
AVS	Adrenal Venous Sampling
CVDs	Cardiovascular Diseases
EETs	Epoxyeicosatrienoic acids
EH	Essential Hypertension
FA	Fatty Acid
FAME	Fatty Acid Methyl Ester
IV-SLT	Intravenous Salt Loading Test
MRA	Mineralocorticoid Receptor Antagonist
MUFA	Monounsaturated Fatty Acid
PA	Primary Aldosteronism
PUFA	Polyunsaturated Fatty Acid
SFA	Saturated Fatty Acid
